# Falling towards Forgetfulness: Synaptic Decay Prevents Spontaneous Recovery of Memory

**DOI:** 10.1371/journal.pcbi.1000143

**Published:** 2008-08-22

**Authors:** James V. Stone, Peter E. Jupp

**Affiliations:** 1 Psychology Department, Sheffield University, Sheffield, United Kingdom; 2 School of Mathematics and Statistics, University of St Andrews, St Andrews, United Kingdom; University College London, United Kingdom

## Abstract

Long after a new language has been learned and forgotten, relearning a few words seems to trigger the recall of other words. This “free-lunch learning” (FLL) effect has been demonstrated both in humans and in neural network models. Specifically, previous work proved that linear networks that learn a set of associations, then partially forget them all, and finally relearn some of the associations, show improved performance on the remaining (i.e., nonrelearned) associations. Here, we prove that relearning forgotten associations *decreases* performance on nonrelearned associations; an effect we call negative free-lunch learning. The difference between free-lunch learning and the negative free-lunch learning presented here is due to the particular method used to induce forgetting. Specifically, if forgetting is induced by isotropic *drifting* of weight vectors (i.e., by adding isotropic noise), then free-lunch learning is observed. However, as proved here, if forgetting is induced by weight values that simply decay or *fall* towards zero, then negative free-lunch learning is observed. From a biological perspective, and assuming that nervous systems are analogous to the networks used here, this suggests that evolution may have selected physiological mechanisms that involve forgetting using a form of synaptic drift rather than synaptic decay, because synaptic drift, but not synaptic decay, yields free-lunch learning.

## Author Summary

If you learn a skill, then partially forget it, does relearning part of that skill induce recovery of other parts of the skill? More generally, if you learn a set of associations, then partially forget them, does relearning a subset induce recovery of the remaining associations? In previous work, in which participants learned the layout of a scrambled computer keyboard, the answer to this question appeared to be “yes.” More recently, we modeled this “free-lunch learning” effect using artificial neural networks, in which the synaptic strength between each pair of model neurons is a connection weight. We proved that if forgetting is induced by allowing each weight value to drift randomly, then free-lunch learning is almost inevitable. However, if, after learning a set of associations, forgetting is induced by allowing each connection weight to *decay* or *fall* toward zero, then relearning a subset of associations decreases performance on the remaining associations. This suggests that evolution may have selected physiological mechanisms that involve forgetting using a form of synaptic drift rather than synaptic decay, because synaptic drift yields free-lunch learning, whereas decay does not.

## Introduction

The idea that structural changes underpin the formation of new memories can be traced to the 19th century [1]. More recently, Hebb proposed that “When an axon of cell A is near enough to excite B and repeatedly or persistently takes part in firing it, some growth process or metabolic change takes place in one or both cells such that A's efficiency, as one of the cells firing B, is increased” [2]. It is now widely accepted that learning involves some form of Hebbian adaptation, and a growing body of evidence suggests that Hebbian adaptation is associated with the long-term potentiation (LTP) observed in neuronal systems [3]. LTP is an increase in synaptic efficacy which occurs in the presence of pre-synaptic and post-synaptic activity, and can be specific to a single synapse. One consequence of Hebbian adaptation is that information regarding a specific association is distributed amongst many synaptic connections, and therefore gives rise to a distributed representation of each association.

In [4], participants learned the layout of letters on a “scrambled” keyboard. After a period of forgetting, participants relearned a subset of letter positions. Crucially, this improved performance on the remaining (i.e., nonrelearned) letter positions. However, whereas relearning some associations shows evidence of FLL in some studies [4]–[6], this is not found in not all studies [7]. This discrepancy may be because the many studies performed to investigate this general phenomenon use a wide variety of different materials and procedures, with some measuring recall and others measuring recognition performance, for example. However, within the realms of psychology, one relevant effect is known as part-set cueing inhibition.

Part-set cueing inhibition [8] occurs when a subject is exposed to part of a set of previously learned items, which is found to reduce recall of nonrelearned items. However, [9] showed that a learned row of words was better recalled if the cues consisted of a subset of words placed in their learned positions than if cue words were placed in other positions. In this case, part-set cueing seems to improve performance, but only if each “part” appears in the spatial position in which it was originally learned. This position-specificity is consistent with the FLL effect reported using the “scrambled keyboard” procedure in [4] but has no obvious concomitant in network models (e.g., [4],[10],[11]).

If the brain stores information as distributed representations, then each neuron contributes to the storage of many associations. Therefore, relearning some old and partially forgotten associations should affect the integrity of other associations learned at about the same time. As noted above, previous work has shown that relearning some forgotten associations does not disrupt other associations, but partially restores them. This FLL effect has also been demonstrated in neural network models ([10],[12]), where it can accelerate evolution of adaptive behaviors [13]. Crucially, in [12], the proof that relearning some associations partially restores other associations assumes that forgetting is caused by the addition of isotropic noise to connection weights, which could result from the cumulative effect of small random changes in connection weights. In contrast, here we prove that if forgetting is induced by shrinking weights towards zero, so that weights “fall” towards the origin, then relearning some associations disrupts other associations.

**Figure 1:**
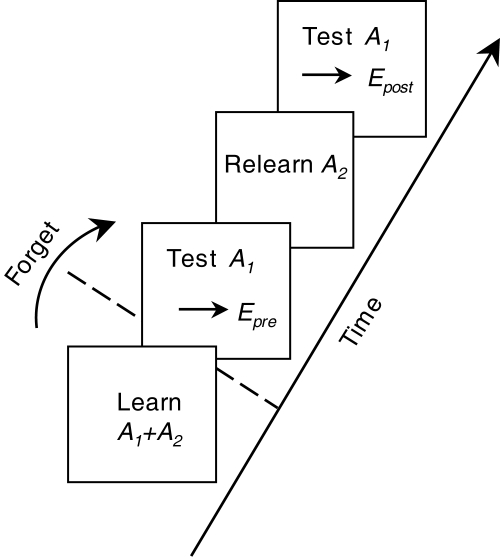
Free-lunch learning protocol. Two subsets of associations *A*_1_ and *A*_2_ are learned. After partial forgetting (see text), performance error *E*_pre_ on subset *A*_1_ is measured. Subset *A*_2_ is then relearned to pre-forgetting levels of performance, and performance error *E*_post_ on subset *A*_1_ is re-measured. If *E*_post_<*E*_pre_ then FLL has occurred, and the amount of FLL is *δ* = *E*_pre_−*E*_post_. Redrawn from [12].

The protocol used to examine FLL here is the same as that used in [4] and [12] and is as follows (see Figure 1). First, learn a set of *n*_1_+*n*_2_ associations *A* = *A*_1_∪*A*_2_ consisting of two subsets *A*_1_ and *A*_2_ of *n*_1_ and *n*_2_ associations, respectively. After all learned associations *A* have been partially forgotten, measure performance error on subset *A*_1_. Finally, relearn *only* subset *A*_2_ and then remeasure performance on subset *A*_1_. FLL occurs if relearning subset *A*_2_ improves performance on *A*_1_.

In order to preclude a common misunderstanding, we emphasize that, for a network with *n* connection weights, it is assumed that *n*≥*n*_1_+*n*_2_ ; that is, the number of connection weights on each output unit is not less than the number *n*_1_+*n*_2_ of learned associations. Using the class of linear network models described below, up to *n* associations can be learned perfectly (see [12]).

The proofs below refer to a network with one output unit. However, these proofs apply to networks with multiple output units, because the *n* connections to each output unit can be considered as a distinct network, in which case our results can be applied to the network associated with each output unit.

### Definition of Performance Error

Each association consists of an input vector **x** and a corresponding target value *d*. For a network with weight vector **w**, the response to an input vector **x** is *y* = **w·x**. We define the *performance error* for input vectors **x**_1_,…,**x** *_k_* and desired outputs *d*_1_,…,*d_k_* to be
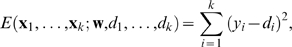
(1)where *y_i_* = **w**·**x** *_i_* is the output response to the input vector **x** *_i_*. By putting **X** = (**x**_1_,…,**x** *_k_*)*^T^*, **d** = (*d*_1_,…,*d_k_*)*^T^* and
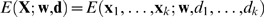
we can write Equation 1 succinctly as
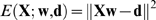
(2)


The two subsets *A*_1_ and *A*_2_ consist of *n*_1_ and *n*_2_ associations, respectively. Let **w**_0_ be the network weight vector after *A*_1_ and *A*_2_ are learned. When *A*_1_ and *A*_2_ are forgotten, the network weight vector changes to **w**_1_, say, and the performance error on *A*_1_ becomes *E*_pre_ = *E*(**X**;**w**_1_,**d**). Finally, relearning *A*_2_ yields a new weight vector, **w**_2_, say, and the performance error on *A*_1_ is *E*_post_ = *E*(**X**;**w**_2_,**d**). Free-lunch learning has occurred if performance error on *A*_1_ is less after relearning *A*_2_ than it was before relearning *A*_2_ (i.e., if *E*_post_<*E*_pre_).

Given weight vectors **w**_1_ and **w**_2_, a matrix **X** of input vectors, and a vector **d** of desired outputs, define
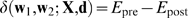
(3)which we shall also refer to simply as *δ*.

In previous work [12], we assumed that the “forgetting vector” **v** (defined as **v** = **w**_1_−**w**_0_) has an isotropic distribution. Here we shall assume instead that the post-forgetting weight vector **w**_1_ is given by
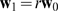
(4)for some (possibly random) scalar *r*, so that
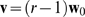
(5)and therefore
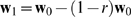
(6)The interpretation of Equation 6 is that forgetting consists of making the optimal weight vector **w**_0_ “fall” towards the origin by a *falling factor* 1−*r*.

## Results

We provide theoretical results, and compare these with results obtained using computer simulations. In essence, our theoretical and simulation results indicate that falling weights induce negative FLL, which decreases with the square of the falling factor 1−*r*.

### Theoretical Results

Our two main theorems are summarised here, and proofs are provided in the Methods section. These theorems apply to a network with *n* weights which learns *n*_1_+*n*_2_ associations *A* = *A*_1_∪*A*_2_, and then after partial forgetting, relearns the *n*_2_ associations in *A*_2_.

We prove that if *n*_1_+*n*_2_≤*n* (so that, in general, the associations *A*_1_ and *A*_2_ are consistent) and the joint distribution of (**X**_1_,**d**_1_) is isotropic (where **X**_1_ and **d**_1_ are the matrix of inputs and the vector of desired outputs for subset *A*_1_ of associations) then the expected value of *δ* is negative (recall that *δ* is defined in Equation 3). We then prove that the probability *P*(*δ*<0) that *δ* is negative approaches unity as *n*_1_ approaches ∞.

### Theorem 1

For every non-zero value of *r*, the expected value of *δ* given *r* is negative. More precisely,
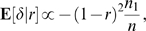
(7)with equality only in trivial cases, and where the constant of proportionality is guaranteed to be positive. Thus, the expected amount of FLL is negative (or zero).

From a physiological perspective, the case *r*<1 is obviously of interest because it represents synaptic weight decay. However, from a mathematical perspective, Theorem 1 applies to every value of *r*, and so it also holds for *r*>1. In other words, any movement of the weight vector **w** along the the line connecting **w**_0_ to the origin yields an expectation of negative FLL, in accordance with Theorem 1.

### Theorem 2

Under mild conditions on the distributions of the input/output pairs (**X**_1_,**d**_1_) and (**X**_2_,**d**_2_),
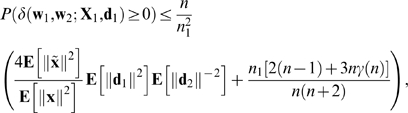
(8)where **x** and 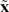 are any columns of 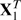 and 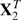, respectively, and
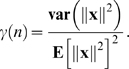



Theorem 2 implies that, if (i) the number (*n*_1_) of associations in *A*_1_ is a fixed non-zero proportion ( *n*_1_/*n* ) of the number *n* of connection weights, (ii) **E**[∥**d**_1_∥^2^]**E**[∥**d**_2_∥^−2^] is bounded as *n* → ∞, and (iii) *γ*(*n*) → 0 as *n* → ∞ then *P*(*δ*>0) → 0 as *n* → ∞, i.e., the amount of FLL is negative, with a probability which tends to 1 as *n* → ∞.

For example, if we assume that (i) each input vector **x** = (*x*_1_,…,*x_n_*) is chosen from an isotropic Gaussian distribution and (ii) the variance of *x_i_* is 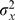 then *γ*(*n*) = 2/*n*, 
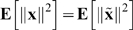
, and **E**[∥**d**_1_∥^2^]**E**[∥**d** ^2^∥^−2^] = *n*_1_/(*n*_2_−1). This ensures that *P*(*δ*>0) → 0 as *n* → ∞.

### Simulation Results

Simulation was carried out on a network with *n* input units and one output unit. The set *A* of associations consisted of *k* input vectors (**x**_1_,…,**x** *_k_*) and *k* corresponding desired scalar output values (*d*_1_,…,*d_k_*). Each input vector comprised *n* elements **x** = (*x*_1_,…,*x_n_*). The values of *x_i_* and *d_i_* were chosen from a Gaussian distribution with unit variance (i.e., 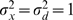). A network's output *y_i_* is a weighted sum of input values 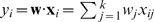, where *x_ij_* is the *j*th component of the *i*th input vector **x** *_i_*, and each weight *w_j_* is the connection between the *j*th input unit and the output unit.

Given that the network error for a given set of *k* associations is 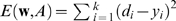, the derivative 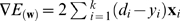 of *E* with respect to **w** yields the delta learning rule 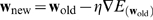, where *η* is the learning rate, which is adjusted according to the number of weights.

However, in order to save time, we used an equivalent learning method. Learning of the *k* = *n* associations in *A* = *A*_1_∪*A*_2_ was performed by solving a set of *n* simultaneous equations using a standard method, after which the weight vector **w**_0_ was obtained; this provided perfect performance on all *n* associations. Partial forgetting was induced by making weights “fall” towards the origin **w**_1_ = *r* **w**_0_, after which performance error was *E*_pre_. Relearning the *n*_2_ = *n*/2 associations in *A*_2_ was implemented with *k* = *n*_2_ as above, after which performance error was *E*_post_.

**Figure 2:**
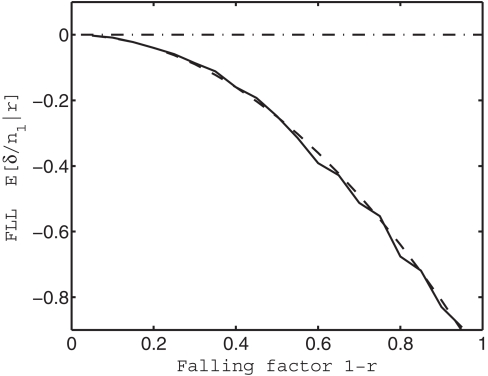
Free-lunch learning decreases as the network's weight vector falls toward the origin. A network with 100 input units and one output unit learns two subsets *A*_1_ and *B*_2_, each of which consists of 50 associations. After learning *A*_1_ and *A*_2_, the network has a weight vector w = w_0_, but after partial forgetting, the weight vector is w = w_1_. If forgetting consists of subtracting a proportion 1−*r* of w_0_ such that w_1_ = w_0_−(1−*r*)w_0_ then the weight vector “falls” towards the origin; the factor 1−*r* is called the *falling factor*. After forgetting, performance error on *A*_1_ is *E*_pre_, an error which changes to *E*_post_ after relearning *A*_2_, where this change is *δ* = *E*_pre_−*E*_post_. Given that there are *A*_1_ associations in *A*_1_, the expected free-lunch learning per association in *A*_1_ is therefore E[*δ*/*n*_1_|*r*]. *Solid curve*: the expected FLL, *E*[*δ*/*n*_1_|*r*], where this expectation is taken over 100 computer simulations. *Dashed curve*: theoretical prediction of *E*[*δ*/*n*_1_|*r*] (see Equation 7), using a constant of proportionality equal to unity, so that the predicted free-lunch learning is *E*_predict_[*δ*/*n*_1_|*r*] = −(1−*r*)^2^. As predicted, free-lunch learning *E*[*δ*/*n*_1_|*r*] becomes more negative as the falling factor 1−*r* increases.

In each simulation, each value in each input vector **x** *_i_*, and each target value *d_i_* was chosen from the same isotropic gaussian distribution with unit variance. There were 100 input units, and one output unit. The subsets *A*_1_ and *A*_2_ each consisted of 50 associations. The value of *δ* = *E*_pre_−*E*_post_ was obtained in each of 100 simulations, using a different random seed for each simulation. In Figure 2, the mean of 100 values of *δ* is shown for various values of the falling factor 1−*r*.

### The Geometry of Forgetting

**Figure 3:**
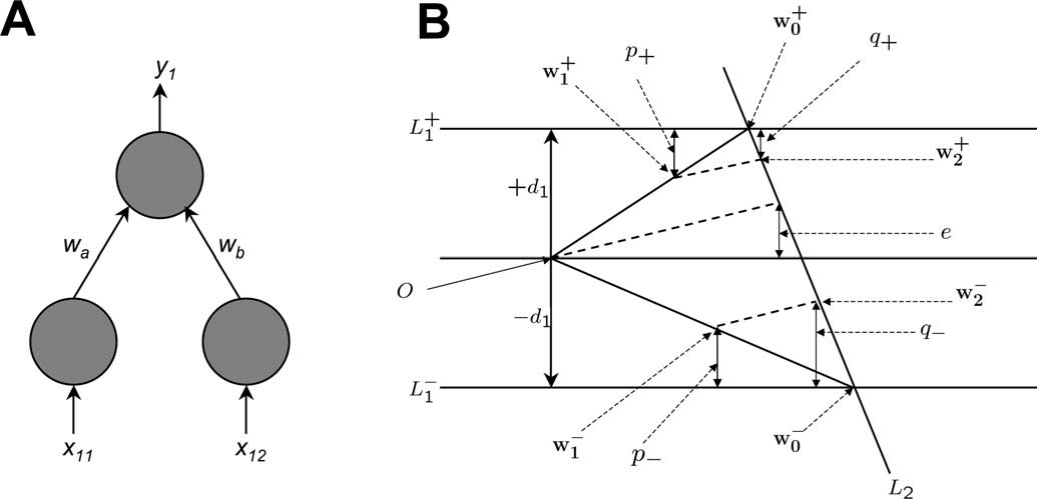
Geometric example of how relearning *A*_2_ increases the error on *A*_1_. (A) A network with two input units and one output unit, with connection weights *ω_a_* and *ω_b_* defines a weight vector w = (*ω_a_*,*ω_b_*). The network learns two associations *A*_1_ and *A*_2_. For example, *A*_1_ is the mapping from input vector x_1_ = (*x*_11_,*x*_12_) to desired output value *d*_1_, and learning *A*_1_ consists of adjusting w until the network output *y*_1_ = w·x_1_ equals *d*_1_. (B) For a given association *A*_2_ = (*X*_2_,*d*_2_), the corresponding constraint line in the space defined by (*ω_a_*,*ω_b_*) is *L*_2_. Irrespective of the precise value of the target output value *d*_1_ in association *A*_1_, if *d*_1_ is distributed isotropically then +*d*_1_ is as probable as −*d*_1_. When averaged over +*d*_1_ and −*d*_1_, the change *δ* in error on *A*_1_ induced by relearning *A*_2_ can be shown to be −(1−*r*)^2^ *e* ^2^, where w_1_^±^ = *r*w_0_^±^. Since this is less than zero, the expected change *E*[*δ*|*r*]<0. (Figure 3A redrawn from [12]).

We present a brief account of the geometry which underpins the results reported here, for a network with two input units and one output unit, as shown in Figure 3A. This network learns two associations *A*_1_ = (*X*_1_,*d*_1_) and *A*_2_ = (*X*_2_,*d*_2_).

Figure 3B provides a geometric example of how relearning *A*_2_ increases the error on *A*_1_. After learning *A*_1_ and *A*_2_, **w** = **w**_0_. The effects of forgetting and relearning can be seen by ignoring the ± superscripts and subscripts for now. After partial forgetting, **w** = **w**_1_, and performance error *E*_pre_ = *p* ^2^. Relearning *A*_2_ yields **w**_2_, the orthogonal projection of **w**_1_ on to *L*_2_, and performance error is *E*_post_ = *q* ^2^. FLL occurs if *δ* = *E*_pre_−*E*_post_>0, or equivalently if *p* ^2^−*q* ^2^>0 (see [12], Appendices A–C for proofs). Forgetting here consists of reducing **w**_0_ by a factor *r*<1, so that **w**_1_ = *r* **w**_0_.

The plus and minus signs in Figure 3B refer to two versions 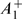 and 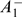 of association *A*_1_, in which *X*_1_ is the same and the target *d*_1_ has the same magnitude, but opposite signs: 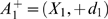 and 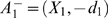.

We now find the expected change in error induced by relearning a given association *A*_2_. After learning 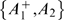 followed by forgetting, the change in error on 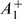 after relearning *A*_2_ is 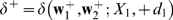. After learning 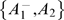 followed by forgetting, the change in error on 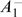 after relearning *A*_2_ is 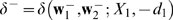. Using similar triangles in Figure 3B,
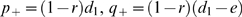
(9)

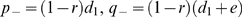
(10)Therefore, the total change in error on 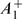 and 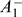 induced by relearning *A*_2_ (on different occasions) is
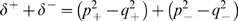
(11)

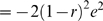
(12)

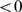
(13)Irrespective of the precise value of the target output value *d*_1_ in *A*_1_, if the distribution of *d*_1_ is isotropic then +*d*_1_ is as probable as −*d*_1_. If the total change in error for two instances (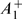 and 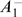) of *A*_1_ is −2(1−*r*)^2^ *e* ^2^ then the expected change (conditional on *e* ) is *E*[*δ*|*e*] = −(1−*r*)^2^ *e* ^2^. Therefore, if forgetting is induced by falling weight values, then the expected change in error **E**[*δ*]<0.

## Discussion

We have proved and demonstrated that, in one of the simplest forms of neural network model, relearning part of a previously learned set of associations reduces performance on the remaining non-relearned associations. This result is in stark contrast to our previous results, which proved that relearning induced partial recovery of non-relearned items [12]. The only difference between these two studies is the way in which forgetting was induced.

An obvious physiological concomitant of Hebbian learning is long-term potentiation (LTP), which seems to underpin learned behaviors [14]. LTP can last for hours, days or even months, and usually follows an exponential decay [3]. However, some forms of LTP do not seem to decay [15], and have been shown to be stable for up to one year [16]. Such stability is remarkable, but from a statistical point of view, would almost certainly be accompanied by random fluctuations which would have a cumulative effect over time; and indeed, fluctuations are apparent in the stable LTP reported in [16]. Crucially, it is not known if the forgetting of learned behaviors is caused by decaying efficacy at many synapses, or by the cumulative effect of random fluctuations in stable LTP-induced synaptic efficacies. Here, decaying efficacy is analogous to weight values that fall toward zero in network models, whereas the cumulative effect of random fluctuations is analogous to the addition of random noise, or drifting, of weight values in network models.

Given a choice between forgetting via synaptic weights that fall towards zero and weights that drift isotropically, has evolution chosen drifting or falling? If all other things were equal then forgetting via synaptic drift would seem to be the obvious choice. This is because drifting ensures that relearning a subset of associations improves performance on other associations, whereas falling decreases performance. However, other things are rarely equal. The expected magnitude of weights increases with drifting but decreases with falling. (Consider a hypersphere centered on the origin, with radius ∥**w**_0_∥ . Simple geometry shows that more than half of all directions emanating from **w**_0_ yield a new weight vector **w**_1_ which lies outside the hypersphere, and therefore **E**[∥**w**_1_∥]>**E**[∥**w**_0_∥] (assuming, for example, that all vectors **w**_1_−**w**_0_ have the same length).) This decrease in weight magnitudes effectively reduces neuronal firing rates, which reduces metabolic costs relative to costs incurred by synaptic drift. Synaptic drift therefore confers mnemonic benefits, but these benefits come at a metabolic price. Thus the increased fitness gained from the mnemonic benefits of synaptic drift must be offset against their metabolic costs. In essence, even free-lunch learning comes at a price.

## Methods

We proceed by deriving expressions for *E*_pre_, *E*_post_, and for *δ* = *E*pre−*E*_post_. We prove that if *n*_1_+*n*_2_≤*n* then the expected value of *δ* is negative. We then prove that the probability *P*(*δ*<0) that *δ* is negative approaches unity as *n*_1_ approaches ∞.

### Performance Errors

Given a *c*×*n* matrix **X** and a *c* -dimensional vector **d**, let *L* **_X_**_,**d**_ be the affine subspace
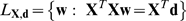
of 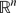. If **X** and **d** are consistent (i.e., there is a **w** such that **Xw** = **d**) then
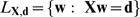
Given weight vectors **w**_1_ and **w**_2_, a matrix **X** of input vectors, and a vector **d** of desired outputs, define
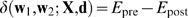
where *E*_pre_ = *E*(**X**;**w**_1_,**d**) and *E*_post_ = *E*(**X**;**w**_2_,**d**). Let 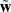 be any element of *L* **_X_**_,**d**_. Then 
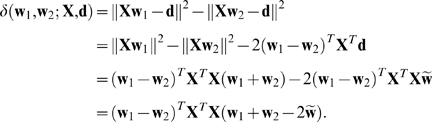
(14)


If **X** *_i_* has rank *n_i_* then transposing the QR decomposition of 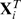 (or, equivalently, using Gram–Schmidt orthonormalisation of the rows of **X** *_i_*) gives
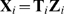
for unique *n_i_*×*n_i_* and *n_i_*×*n* matrices **T** *_i_* and **Z** *_i_* with **T** *_i_* lower triangular with positive diagonal elements, and 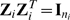. Simple calculation shows that, for any weight vector **w**, 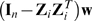 and 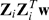 are orthogonal. Since 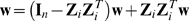, it follows that the matrix 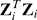 represents the operator that projects orthogonally onto the image of 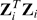. Because
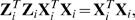
(15)the image of 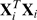 is contained in that of 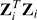. As both these images have dimension *n_i_*, they must be equal, and so 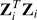 represents the operator which projects orthogonally onto the image of 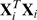.

Now suppose that **X** and **d** are consistent, where
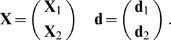



Then, after the network has learned *A*_1_ and *A*_2_, the weight vector **w**_0_ satisfies
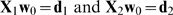
(16)(If, as below, *n*_1_+*n*_2_≤*n*, **X**_2_ and **d**_2_ are consistent, and (**X**_1_,**d**_1_) has a continuous distribution then Equation 16 holds with probability 1.)

### Falling

We now assume that forgetting is induced by weight values “falling” towards the origin at zero, i.e., forgetting consists of shrinking the weight vector **w**_0_ by a (possibly random) factor *r* towards the “dead state” **0**. Thus the post-forgetting weight vector **w**_1_ is given by
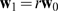
(17)and so the “forgetting vector” **v** = **w**_1_−**w**_0_ is
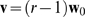
(18)


The form of forgetting given by Equation 17 is very different from that investigated in [12], where **v** has an isotropic distribution and is independent of (**X**_1_,**d**_1_) and (**X**_2_,**d**_2_).

Let **w**_2_ be the orthogonal projection of **w**_1_ onto *L*_2_. Then
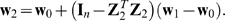



Manipulation gives
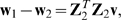
(19)and so
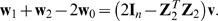
(20)


Then Equations 14, 16, and 18–20 yield
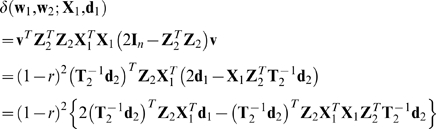
(21)


### The Case of Isotropic Random (**X**_1_,**d**_1_)

In this section we assume that the distribution of (**X**_1_,**d**_1_) is isotropic, i.e., that (**UX**_1_ **V**,**Ud**_1_) has the same distribution as (**X**_1_,**d**_1_) for all orthogonal *n*_1_×*n*_1_ matrices **U** and all orthogonal *n*×*n* matrices **V**. Then taking the conditional expectation of Equation 21 for given **X**_2_, **d**_2_, and *r* gives the following theorem.

### Theorem 1

If

1. *n*_1_+*n*_2_≤*n*,
2. **X**_2_ and **d**_2_ are consistent,
3. the distribution of (**X**_1_,**d**_1_) is continuous and isotropic,
4. **X**_1_, **d**_1_, and (**X**_2_,**d**_2_,*r*) are independent.

then
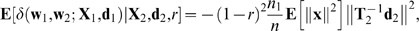
(22)where **x** is any column of 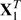.

### Corollary 1

If 1.-3. of Theorem 1 hold then
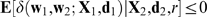
(23)with equality if and only if either *r* = 1 or **d**_2_ = 0.

Corollary 1 says that (apart from trivial exceptions) the expected amount of FLL is negative.

To obtain Theorem 2, it is useful to have some moments of isotropic distributions. Let **x** be isotropically distributed on 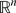. Then Equations 9.6.1 and 9.6.2 of Mardia and Jupp (2000), together with some algebraic manipulation, yield
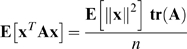
(24)

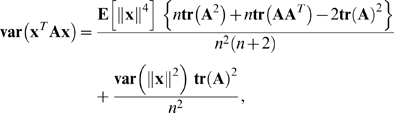
(25)as in Equations A.14 and A.15 of [12].

The other tool used in proving Theorem 2 is the formula
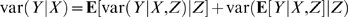
(26)for any random variables *X*,*Y*,*Z* for which these quantities exist. Equation 26 is an application to the conditional distribution of *Y*|*Z* of the standard conditional variance formula that is given in Equation 2b.3.6 on page 97 of [17].

Taking the expectation and variance of Equation 21 as only **d**_1_ varies and using Equation 24 gives
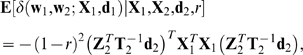
(27)

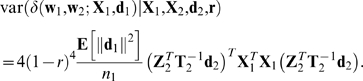
(28)


Taking the expectation of Equation 28 as only **X**_1_ varies and using Equation 24 gives
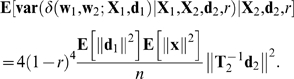
(29)


We now suppose that
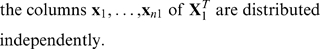
(30)


Then taking the variance of Equation 27 as only **X**_1_ varies and using Equation 25 gives
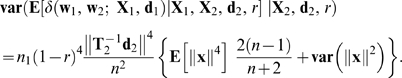
(31)


Adding Equations 29 and 30 and using Equation 26 yields
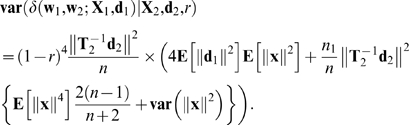
(32)


To obtain an upper bound on the conditional probability of FLL (i.e., on *P*(*δ*≥0|**X**_2_,**d**_2_,*r*)), we use Chebyshev's inequality, which states that, for any random variable *Y* and any positive value of *t*

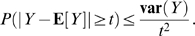



Applying Chebyshev's inequality to the conditional distribution of δ(**w**_1_,**w**_2_,**X**_1_,**d**_1_) given (**X**_2_,**d**_2_,*r*), taking *t* = **E**[*δ*(**w**_1_,**w**_2_;**X**_1_,**d**_1_)|**X**_2_,**d**_2_,*r*], and noting that (by Equation 23) *t*≤0, we obtain
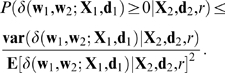
(33)


Substituting Equations 22 and 32 into Equation 33 gives
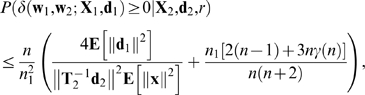
(34)where
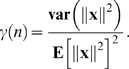



For any positive-definite symmetric matrix **A** and vector **x**, diagonalization of **A**, together with the fact that *x*+1/*x*≥2 for positive *x*, yields
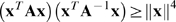
(35)


Combining Equations 34 and 35 with the fact that 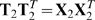 gives
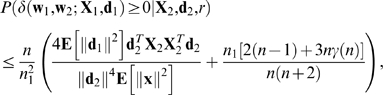
(36)


Taking the expectation of Equation 36 over **X**_2_ yields
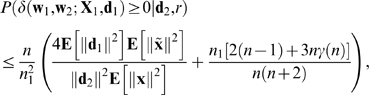
(37)where **x** and 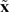 are any columns of 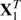 and 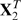, respectively.

Taking the expectation of Equation 37 over **d**_2_ and *r* yields the following theorem.

### Theorem 2

If (a) conditions 1.-4. of Theorem 1 hold, (b) the columns 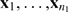 of 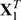 are distributed independently, (c) **X**_2_, **d**_2_, and *r* are independent, (d) the distribution of (**X**_2_,**d**_2_) is isotropic, and (e) **E**[∥**d**_2_∥^−2^] is finite then
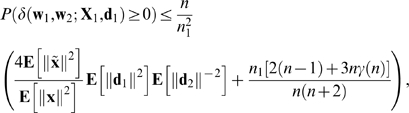
(38)where **x** and 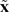 are any columns of 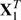 and 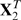, respectively, and
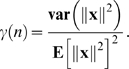



### Corollary 2

If the conditions of Theorem 2 hold and
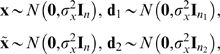
where **x** and 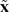 are any columns of 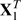 and 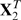, respectively, then
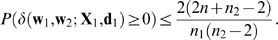



Thus
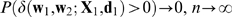
provided that *n*_1_/*n* and *n*_2_/*n* are bounded away from zero.
